# Role of the skin microbiota and intestinal microbiome in rosacea

**DOI:** 10.3389/fmicb.2023.1108661

**Published:** 2023-02-10

**Authors:** Weitao Zhu, Michael R. Hamblin, Xiang Wen

**Affiliations:** ^1^Clinical Medicine (Eight-Year Program), West China School of Medicine, Sichuan University, Chengdu, China; ^2^Laser Research Centre, Faculty of Health Science, University of Johannesburg, Doornfontein, South Africa; ^3^Department of Dermatology, West China Hospital, Sichuan University, Chengdu, China

**Keywords:** rosacea, skin microbiota, gastrointestinal microbiome, influence factors, treatment

## Abstract

Rosacea is a chronic inflammatory cutaneous disorder of uncertain etiology that mainly affects the centrofacial region, including cheeks, nose, chin, forehead, and eyes. The pathogenesis of rosacea remains unclear because it involves several complex factors. Additionally, the potential treatment methods need to be explored. We reviewed the common bacterial species in the skin microbiota and gut microbiota of rosacea patients such as *Demodex folliculorum*, *Staphylococcus epidermidis*, *Bacillus oleronius*, *Cutibacterium acnes*, and *Helicobacter pylori* and identified their role in the pathogenesis. Besides, we summarized the influence factors such as temperature and age on rosacea patients. We also systematically reviewed the commonly used clinical treatment methods, including antibiotics, probiotics. as well as their treatment mechanism and application precautions.

## Introduction

**Figure 1:**
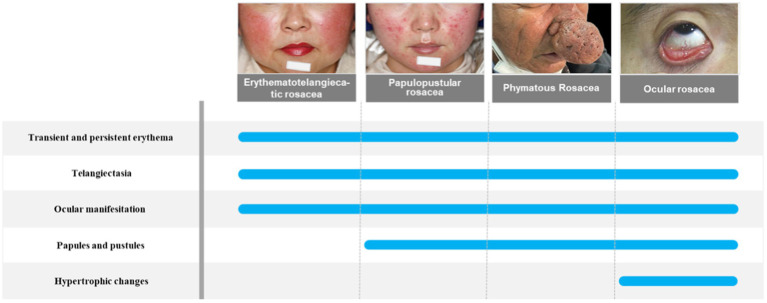
The classification of rosacea.

Rosacea is a chronic inflammatory cutaneous disorder of uncertain etiology that mainly affects the centrofacial region, including cheeks, nose, chin, forehead, and eyes. There are four subtypes of rosacea, which are erythematotelangiectatic rosacea, papulopustular rosacea, phymatous rosacea, and ocular rosacea (Wilkin et al., 2002). However, these subtypes can progress from one type to another, so the current clinical recommendation is to classify rosacea according to clinical presentation, as patients with rosacea can have different clinical signs and symptoms. The newest research has classified rosacea symptoms into recurrent flushes or transient erythema, persistent erythema, morphological changes, papules, pustules, and telangiectasia (van Zuuren et al., 2021). The pathogenesis of rosacea involves several complex factors. Not only genetic factors but also environmental factors have been linked to rosacea. There are several flare triggers in patients with rosacea, including temperature changes, heat, cold, exercise, ultraviolet radiation, spicy food, and alcohol (Buddenkotte and Steinhoff, 2018). These factors can make patients more susceptible to skin disorders because they alter the skin’s epidermal barrier function or disrupt immune function (Park et al., 2021). Rosacea is associated with many systemic complications such as gastrointestinal disease, cardiovascular disease, neurological disease, psychiatric disease, and autoimmune disease, but the exact pathogenesis of rosacea remains unclear (Holmes et al., 2018; Figure 1). The classification of rosacea is shown in Figure 1.

In the pathogenesis of rosacea, there has been extensive discussion on the skin microbiota and its related inflammatory effects. Many different communities of microorganisms have been studied in the skin, formed by hundreds of microbial species occupying different environmental niches in the skin (Xu and Li, 2019). The skin microbiota is essential for regulating inflammation and immune responses. The epidermis, dermis, and deeper subcutaneous tissue together form a physical and chemical barrier against external pathogens (Chen et al., 2021). Temporary non-specific immune cells and highly specific long-acting immune components constitute the skin immune barrier (Chaplin, 2010). The bacteria, fungi, viruses, and arthropods that live on the human skin together make up the human skin microbiome, all of which have been found to play a role in regulating immune responses. Some of these can cross the skin barrier and interact with deeper cells. If the skin microbiome is disturbed by internal or external factors, it can interfere with the function of the immune barrier to maintain homeostasis. Microorganisms in the skin not only trigger the release of certain antimicrobial peptides, but also regulate components of the complement system, and aggravate skin inflammation by accumulating neutrophils and producing interleukins (Park and Lee, 2018). However, the skin is not only affected by its own microorganisms, because recent studies have suggested that the skin can be affected by the gastrointestinal microbiome. The most frequently mentioned comorbidity is gastrointestinal disease among all kinds of rosacea. It has been gradually recognized that commensal microbes may play a significant part in the development of certain cutaneous disorders, and it is also believed that a weakened external barrier to pathogens leads to dysregulation of the skin microecology (Lam et al., 2022). Therefore, in this review, we summarize reports about the association between rosacea and the skin microbiota and gastrointestinal microbiota and provide an overall picture of the impact of rosacea treatment on the skin and gut microbiota.

## Studies of the skin microbiome of patients with rosacea

Like most organ systems, the microbiota within the skin is indispensable for promoting efficient immune function. Researchers have identified several microbes as potential contributors to the development of rosacea; these are *Demodex folliculorum*, *Staphylococcus epidermidis*, *Bacillus oleronius*, and *Cutibacterium acnes* (Holmes, 2013).

*Demodex folliculorum* are microscopic mites which are usually found at the base of the eyelashes. The adult mites are cigar-shaped with four legs to grasp cylindrical structures like eyelashes. Demodex infection can cause activation of the immune system, inflammation, and follicular changes that may lead to disease (Fromstein et al., 2018).

*Staphylococcus epidermidis* is a Gram-positive biofilm-producing symbiotic bacteria and is the most important member of coagulase-negative staphylococci, widely present on human skin and mucosa, *S. epidermidis* is one of the most abundant colonizers on human skin. It could attach to foreign objects and form biofilms, which contributes to its ability to cause infectious disease (Yuan et al., 2020).

The *Bacillus* genus is a group of Gram-positive rod-shaped bacteria that can produce endospores under adverse conditions, making them widespread in nature. Bacillus species include some pathogens of clinical interest, bacterial contaminants in food, and some are used as industrial organisms to produce various enzymes (Owusu-Darko et al., 2017).

*Cutibacterium acnes* is a lipophilic anaerobic Gram-positive bacterium belonging to the *Cutibacterium* spp. family. It is a part of the skin commensal flora and is generally found in hair follicles and sebaceous glands, and can also exist in the oral mucosa, nose, urogenital tract, and large intestine (Achermann et al., 2014).

Demodex mites are associated with the presence of other microbiota in the skin. *Firmicutes*, *Actinobacteria*, and *Proteobacteria* were the most represented phyla in these Demodex related microbiota. Studies comparing rosacea patients with healthy standardized skin surface biopsies to study Demodex-associated microbiota, reported that *Proteobacteria* and *Firmicutes* were more abundant at the phylum level, whereas actinobacteria were less abundant (Murillo et al., 2014). By analyzing the microbial β-diversity, the researchers found that the patient-to-sample cluster was less pronounced, while the treatment-to-sample cluster was least pronounced. *Staphylococcus*, *Cutibacterium*, *Pseudomonas*, *Corynebacterium*, *Acinetobacter*, and *Snodgrasella* were the main bacterial groups at the genus level in untreated rosacea patients (Tutka et al., 2020). *Keratomyces acnes* (Rainer et al., 2020) and *S. epidermidis* (Woo et al., 2020b) are the most diverse bacteria on the skin of patients with rosacea.

When focused on the species level, *S. epidermidis* was the most common bacterial species, followed by *Stenotrophomonas rootophilus*, *C. acnes*, and *Corynebacterium tuberculostearicum* (Woo et al., 2020b). Previous studies had revealed diversity in the microbiota among different subtypes of rosacea. The phylum profile in papulopustular rosacea microbial communities was significantly different from erythematotelangiectatic rosacea. *Actinomycetes* accounted for only about one tenth of all clones in the papulopustular rosacea community, while most clones were found in erythematotelangiectatic rosacea. On the other hand, the proportions of *Proteobacteria* and *Firmicutes* in papulopustular rosacea communities were increased compared with erythematotelangiectatic rosacea (Murillo et al., 2014).

Many studies have shown that the innate immune system is aberrantly activated by some skin microorganisms through Toll-like receptor 2 (TLR 2). After TLR 2 expression, antimicrobial peptides can be abnormally produced, and the expression and activity of serine kallikrein were also increased (Picardo and Ottaviani, 2014). Furthermore, TLR 2 can elicit erythema, telangiectasia, and infammation *via* expression of cytokines, chemokines, proteases, and pro-angiogenic factors (van Zuuren et al., 2021). Moreover, rosacea skin evidently showed increased cathelicidin expression, which was expressed by leukocytes as well as epithelial cells, compared to normal skin. This can lead to several unwanted downstream effects such as leukocyte chemotaxis, vasodilatation, angiogenesis, and extracellular matrix deposition (Weiss and Katta, 2017). At the same time, these effects may eventually lead to the development of a long-lasting non-infectious skin condition. *C. acnes* may play a role in protecting healthy skin (Barnard et al., 2020). It could prevent other microorganism from colonizing the skin because it breaks down sebum into free fatty acids (Marples et al., 1971).

The skin microbiome is a variable phenomenon, that alters with age, sex, environmental factors, and the use of cosmetics and antibiotics. There are differences in the pathogenesis of papules and pustules between acne and rosacea, which have been shown to be caused by age affecting the skin microbiome. Some studies have suggested that the severity of rosacea increases with age (Woo et al., 2020b). Under different temperature conditions, members of the normal skin microbiota that do not normally cause disease, such as *S. epidermidis,* can replicate at different rates and can also secrete more virulence factors (Dahl et al., 2004). *Staphylococcus epidermidis* strains isolated from the skin of rosacea patients were found to produce more protein at 37°C than at 30°C. Research has suggested that sudden changes in temperature can lead to worsening rosacea symptoms. The increased mobility and survival of *Demodex mites* at higher temperatures may explain that heat contributes to the worsening of rosacea (He et al., 2018). Bacteria behave differently at varying temperatures and produce different bacterial products. Skin temperature is likely to influence the activity of other skin microbiota, such as aerobic bacteria, anaerobic bacteria, and *Demodex mites*.

## Study on the gastrointestinal microbiota of patients with rosacea

The human gut, like the skin, is home to countless microbes. Intestinal bacterial species such as *Lactobacillus*, *Escherichia coli*, *Bifidobacterium,* and *Streptococcus thermophilus* help to maintain human health, while others are more likely to cause disease, such as *Clostridium difficile*, *Campylobacter*, *Enterococcus faecalis*, and *Helicobacter pylori*.

Probiotics are living beneficial microbial species, but one way for a host to provide useful substrates for probiotic bacteria is the consumption of prebiotics, for example, foodstuffs or supplements containing certain saccharides (fructose, glucose, galactose, inulin, lactulose, sorbitol, or xylitol), These compounds can affect the intestinal microbiota and improve the environment of the skin, by increasing the number of beneficial gut microbes (Szántó et al., 2019).

*Helicobacter pylori* colonizes the human stomach and duodenum and is a microaerophilic Gram-negative bacterial species (Zeng et al., 2015). It can lead to a lifelong infection that is difficult to eradicate and may infect more than half of the human population worldwide. *Helicobacter pylori* can produce cytotoxins and cause gastric mucosal inflammation by proliferating and producing nitric oxide. It can alter physiological processes such as vasodilation, inflammation, and immune regulation (Mahmud et al., 2022). Rosacea is also associated with *H. pylori* seropositivity (Holmes, 2013). One mechanism for this theoretical association has been suggested to be that *H. pylori* can cause skin inflammation and flushing by the activity of cytotoxins and gastrin (Holmes, 2013), while other mechanisms have also been proposed. An autoimmune mechanism involving cross-reactive antibodies has also been hypothesized. This is based on systemic effects due to increased mucosal permeability to digestive tract antigens, or impaired vascular integrity (Wedi and Kapp, 2002). *Helicobacter pylori* infection has been found to be a risk factor for rosacea, but the association between them is weak. However, researchers reported there was a strong association between a positive C13-urea breath test and rosacea, and the C13-urea breath test is accepted as high diagnostic value for *H. pylori* infection (Jørgensen et al., 2017). This may be due to differences in the way *H. pylori* was diagnosed in the past. Besides, various strains of *H. pylori* have different virulence factors, which might lead to the divergence in the reported results (Woo et al., 2020a). Studies have also linked rosacea to overgrowth of various bacteria in the small intestine (Woo et al., 2020b).

A recent concept called the gut-skin axis has been proposed to explain the pathogenesis of many chronic inflammatory disorders, which proposes that skin homeostasis and allostasis are influenced by gastrointestinal health, through a complicated interplay between the immune system, metabolic system, and nervous systems (O'Neill et al., 2016). The gut microbiome has a bidirectional regulatory effect on host immunity, which is considered the primary regulator of the gut-skin axis (Forbes et al., 2016). Disturbances in the gut microbiome could affect the equilibrium of the immune system.

Some studies have analyzed the composition of the gut microbiota and found that there are significant differences between rosacea patients and control groups (Nam et al., 2018). There is ongoing debate about the effect of digestive diseases on rosacea. In rosacea patients’ intestinal bacterial overgrowth, irritable bowel syndrome and chronic inflammatory bowel disease may be more common (Daou et al., 2021). One study found that altered levels of the mammalian synthetic AMP pheromone, plantaricin A could also play a part in rosacea (Nakatsuji and Gallo, 2012).

## The link between skin microbiota and gastrointestinal microbiome

A complicated link between the alimentary tract, brain and skin has been recognized because patients have been found to improve their skin conditions after oral consumption of probiotics or prebiotics, but researchers have yet to thoroughly investigate the link (Tan-Lim et al., 2021). Changes in gastrointestinal microecology are often accompanied by the diagnosis of psychological disorders such as depression and anxiety. It is known that various neurotransmitters or neuropeptides can be induced by psychological stressors (Salem et al., 2018). This may increase intestinal permeability and therefore lead to enteric and systemic inflammation.

The activation of the plasma kallikrein–kinin system could also be influenced by intestinal bacteria (Kendall, 2004). Researchers have reported the increased stimulation of the plasma kallikrein–kinin system in patients with intestinal inflammation and rosacea (Parodi et al., 1980).

## Impact of treatments on the cutaneous and gut microbiome

Treatment for rosacea usually involves education, including avoiding ultraviolet light exposure, extreme temperatures, diet and alcohol. In addition, skin-irritating cosmetics should be avoided and daily use of sunscreen is recommended because ultraviolet exposure can cause severe effects on the skin. Studies have suggested that the signs and symptoms of rosacea should be treated based on the patient phenotype. For individual major symptoms such as transient and persistent erythema, inflammatory papules or pustules, telangiectasia, or lumps, a first-line treatment followed by a general skin-care regimen should be recommended. Several first-line treatments are listed as follow. Transient erythema: α-adrenergics (topical) and beta blockers (oral). Persistent erythema: brimonidine (topical), IPL and PDL. Inflammatory papules/pustules: azelaic acid (topical), ivermectin (topical), doxycycline (oral) and metronidazole (topical). Telangiectasia: electrodessication, IPL, and lasers. Phyma: doxycycline (oral) and Isotretinoin (oral). If there are multiple symptoms in a single patient, a variety of drugs could be used simultaneously to treat them. If treatment is unsatisfactory within a certain period, another treatment, or the addition of another first-line drug is recommended. The type of treatment and the patient’s preference determine whether to continue treatment (Schaller et al., 2017).

Facial erythema can be treated with topical β-blockers or 2-epinephrine agonists, while oral β-blockers have also been shown to be effective (Logger et al., 2020). In severe infections which oral antibiotics have failed to improve, or which relapse after discontinuation of antibiotics, oral low-dose isotretinoin therapy could be effective. Research has suggested that bacteria sensitive to antibiotics may directly or indirectly cause papules and pustules (Dahl et al., 2004). Antibiotic treatment makes the disease less severe and increases the amount of *Weissella confusa*, a potentially beneficial microbe (Ferček et al., 2021). Studies have found that when rosacea is treated with topical or systemic antibiotics, papules and pustules tend to disappear rapidly. Papules and pustules also disappear rapidly when patients are treated with a range of chemically different antibiotics. Treatment can include erythromycin, clindamycin, ampicillin, metronidazole, clarithromycin, and any of the sulfonamides. The apparent disappearance of papules and pustules in patients treated with chemically different antibiotics suggests that bacteria do play a role in the pathogenesis (Dahl et al., 2004). In patients with rosacea, abnormalities in the hair follicles or the microenvironment of the skin surface can lead to worsening disease (Dahl et al., 2004). Coagulase-negative *staphylococci* produce and secrete proteins in the skin or follicles of patients with rosacea, which may lead to increased inflammation and to papules, pustules and dermatitis.

Many dermatologists treat rosacea patients with papules and pustules with topical or systemic antibiotics. Systemic antibiotics must be used continuously in patients with numerous papules and pustules. The anti-inflammatory activity of systemic antibiotics can lead to the disappearance of papules and pustules in rosacea patients.

Tetracycline has several mechanisms of action, such as antibacterial activity, regulation of innate immunity, inhibition of proinflammatory mediators and protease enzymes, etc. However, it is unclear which is the most relevant mechanism for the eliminatiopapules or pustules. Current studies suggest that an imbalance in the intestinal microbiota can lead to inflammatory skin diseases. Because intestinal bacteria may lead to disturbed immune responses, the use of oral metronidazole treatment can improve both inflammatory enteritis and rosacea symptoms (Vera et al., 2018).

Both minocycline and doxycycline were found to treat rosacea with similar results. Minocycline is a broad-spectrum antibiotic used to treat skin infections caused by many bacteria. The most common non-cutaneous adverse event in the treatment of rosacea with minocycline was viral upper respiratory tract infection, while the most common cutaneous adverse event was pruritus (Martins et al., 2021). Studies found that the skin microbiome α-diversity of rosacea patients treated with oral doxycycline was basically the same before and after systemic antibiotic treatment (Woo et al., 2020b). After treatment of rosacea with doxycycline for six weeks, there was a significant increase in the abundance of a bacterium called *Weissella confusa*. Between rosacea subjects and healthy controls, the researchers found that gut microbiome α-diversity was basically the same (Nam et al., 2018). When it came to the diversity of gut microbiota samples, their results were also the same. In one recent study, treatment with doxycycline significantly reduced the severity of rosacea and the number of inflammatory papules or pustules. Doxycycline (40 mg orally) was as effective as minocycline (100 mg orally) and there was no difference in the rate of adverse events (van Zuuren et al., 2019). Delayed release doxycycline 40 mg MR was as effective as 100 mg, with fewer side effects (Del Rosso et al., 2008). Several reports have used sub-antimicrobial doses of doxycycline hyclate 20 mg (SDD). One study used 20 mg of SDD twice daily for eight weeks to treat 50 patients with various stages of rosacea. On average, the inflammatory lesions were reduced by 80% to 100% and the erythema was reduced by 50% (Bikowski, 2003).

Some studies have shown that 0.75% metronidazole gel can be used as a first-line topical treatment for the treatment of rosacea. Researchers used 0.75% metronidazole gel twice a day for 12 weeks in the treatment of rosacea and found that inflammatory lesions and erythema were significantly improved, by 79% for papules and 94% for pustules (Miyachi et al., 2022). Reactive oxygen species and oxidative stress are closely associated with a range of skin conditions. Topical metronidazole can both reduce the production of reactive oxygen species and exert its efficacy in rosacea related diseases through anti-inflammatory and immunomodulatory pathways.

Topical 1% ivermectin can effectively reduce *Demodex mite* density and had a significant effect on rosacea (Ebbelaar et al., 2018). It could also be observed under reflectance confocal microscopy that *Demodex follicularis* would undergo morphological changes through the action of ivermectin, such as “phantom mites.” Mite density decreased significantly after treatment and clinical improvement. Topical permethrin, benzyl benzoate and crotamine have also been shown to affect *Demodex* populations (Forton and De Maertelaer, 2020). Studies have been conducted to treat rosacea with 1% ivermectin cream once daily. Of 910 participants who received ivermectin, 615 showed improvement, with a post-treatment improvement rate of 68% (van Zuuren et al., 2019). Benzyl benzoate and crotamiton have also been shown to be effective.

The long-term use of broad-spectrum antibiotics can lead to the emergence of resistant strains, more adverse events and compliance problems. Sarecycline is a novel tetracycline derivative with narrow spectrum activity targeting Gram-positive bacteria, especially *Bacillus acnes* (Bunick et al., 2021). In a 12-week study of 72 subjects who received oral administration of sarecycline once daily according to body weight, the results showed that sarecycline was effective in treating papules and pustules in adults with rosacea, with an efficacy of 80% (Rosso et al., 2021).

Although rosacea can be treated with effective oral or topical antibiotics, sulfur compounds can change the facial microbiota (van Zuuren et al., 2015) and there is no conclusive evidence that these changes in the skin microbiota are effective in treating the disease. The effects of antibiotic treatment on the gut microbiota are both short-term and long-term. Although antibiotic treatment may be effective in the short term, most skin diseases are associated with long-term disturbances in the microbiota, so this treatment strategy may not be optimal (de Gunzburg et al., 2018).

Some studies have found that topical application of probiotics could directly affect the skin microbiota and immune response (Yu et al., 2020). The effect of topical probiotics on various skin conditions has not been fully explored. Topical and oral probiotics have both been shown to be effective in treating some local diseases. Besides, a combination of topical and oral probiotic treatment may be the most effective (Knackstedt et al., 2020). In general, treatment with probiotics may improve the skin barrier function, reduce inflammation, and reduce the dysregulation of the skin microbiome by restoring a healthy balance of cytokines. For example, TLR2 may be upregulated in rosacea and could be a possible target for probiotics (Tripathi et al., 2019). Besides, oral probiotics can regulate the intestinal microfora and indirectly affect cutaneous conditions (Yu et al., 2020). The consumption of *Bifidobacteria* and *Lactobacillus* to affect the gut can also be used to treat certain cutaneous conditions (Hacini-Rachinel et al., 2009). *Bacillus subtilis* produces spores to colonize the gastrointestinal tract and alter the mucosal barrier microbiome, thereby eradicating *H. pylori* to reduce rosacea symptoms and associated gastrointestinal problems (Pinchuk et al., 2001). The microorganisms in the intestinal microbiome and skin microbiota described in this review are shown in Table 1.

**Table 1:** Some microorganisms closely related to rosacea in intestinal microbiome and skin microbiota.

Authors	Microbes in skin microbiota	Microbes in intestinal microbiota
Holmes (2013)	*Demodex folliculorum Staphylococcus epidermidis Bacillus oleronius Cutibacterium acnes*	*Helicobacter pylori*
Fromstein et al. (2018)	*Demodex folliculorum*	
Yuan et al. (2020)	*Staphylococcus epidermidis*	
Owusu-Darko et al. (2017)	*Bacillus oleronius*	
Achermann et al. (2014)	*Cutibacterium acnes*	
Murillo et al. (2014)	*Firmicutes Actinobacteria Proteobacteria*	
Tutka et al. (2020)	*Staphylococcus Cutibacterium Pseudomonas Corynebacterium Acinetobacter Snodgrasella*	
Rainer et al. (2020)	*Keratomyces acnes*	
Woo et al. (2020a)	*Staphylococcus epidermidis Stenotrophomonas rootophilus C. acnes Corynebacterium tuberculostearicum*	
Barnard et al. (2020)	*Cutibacterium acnes*	
Dahl et al. (2004)	*Staphylococcus epidermidis*	
He et al. (2018)	*Staphylococcus epidermidis Demodex mites*	
Zeng et al. (2015)		*Helicobacter pylori*
Mahmud et al. (2022)		*Helicobacter pylori*
Jørgensen et al. (2017)		*Helicobacter pylori*
Woo et al. (2020b)		*Helicobacter pylori*
Bunick et al. (2021)	*Bacillus acnes*	
Hacini-Rachinel et al. (2009)		*Bifidobacteria Lactobacillus*
Pinchuk et al. (2001)		*Bacillus subtilis Helicobacter pylori*

## Conclusion

Human skin provides a suitable environment for the growth of both beneficial and pathogenic bacteria. It has been shown that rosacea is associated with disturbances in the microbiome of the skin and gut. Therefore, treating rosacea with antibiotics or microbiome modulation has been an attractive approach to disease management. Most dermatologists treat rosacea patients with papules and pustules with topical or systemic antibiotics. Thus, research on changes in the skin and gut microbiota in rosacea patients could contribute to a better understanding of the development and prognosis of the disease.

The role of the gut microbiota in the pathogenesis of rosacea should be further explored. In future studies, the relative abundance of microbial distribution at the strain level will need to be analyzed and different DNA sequencing techniques will need to be used to confirm the various findings. In addition, the clinical complications of rosacea often occur and the pathogenesis and treatment of complications still needs to be further explored, to better manage this disease.

## Author contributions

WZ contributed to data acquisition, analysis, data interpretation, and manuscript drafting. XW contributed to data acquisition, analysis, supervised the review, and revised the manuscript for intellectual content. MH critically edited the article for content and presentation. All authors contributed to the article and approved the submitted version.

## Funding

National Natural Science Foundation of China (81903226). MH was supported by US NIH Grants R01AI050875 and R21AI121700.

## Conflict of interest

MH declares the following potential conflicts of interest. Scientific Advisory Boards: Transdermal Cap Inc., Cleveland, OH; Hologenix Inc. Santa Monica, CA; Vielight, Toronto, Canada; JOOVV Inc., Minneapolis-St. Paul MN; Sunlighten, Kansas City, MO; Consulting; USHIO Corp, Japan; Sanofi-Aventis Deutschland GmbH, Frankfurt am Main, Germany; Klox Asia, Guangzhou, China. Stockholding: Niraxx Light Therapeutics, Inc., Irvine CA; JelikaLite Corp, New York NY.

The remaining authors declare that the research was conducted in the absence of any commercial or financial relationships that could be construed as a potential conflict of interest.

## Publisher’s note

All claims expressed in this article are solely those of the authors and do not necessarily represent those of their affiliated organizations, or those of the publisher, the editors and the reviewers. Any product that may be evaluated in this article, or claim that may be made by its manufacturer, is not guaranteed or endorsed by the publisher.
